# Dual solutions of magnetized radiative flow of Casson Nanofluid over a stretching/shrinking cylinder: Stability analysis

**DOI:** 10.1016/j.heliyon.2024.e29696

**Published:** 2024-04-15

**Authors:** Azhar Mustafa Soomro, Mustafa Abbas Fadhel, Liaquat Ali Lund, Zahir Shah, Mansoor H. Alshehri, Narcisa Vrinceanu

**Affiliations:** a KCAET Khairpur Mir's, Sindh Agriculture University, Tandojam, Sindh, 70060, Pakistan; b Mathematics Department, College of Education for Pure Sciences, University of Al-Muthanna, Samawa, 66001, Iraq; c Department of Mathematical Sciences, University of Lakki Marwat, Lakki Marwat, 28420, Khyber Pakhtunkhwa, Pakistan; d Department of Mathematics, College of Science, King Saud University, P.O. Box 2455, Riyadh, 11451, Saudi Arabia; e Faculty of Engineering, Department of Industrial Machines and Equipments, "Lucian Blaga" University of Sibiu, 10 Victoriei Boulevard, Romania

**Keywords:** Casson nanofluid, MHD, Thermal radiation, Dual solutions, Stability analysis

## Abstract

The enhanced thermal efficiency exhibited by Casson nanofluids offers significant practical applications across various industrial and engineering sectors. This study focuses on the mathematical investigation of the steady magnetohydrodynamic (MHD) boundary layer flow of Casson nanofluid through a stretched/shrinking cylinder, taking into account the effects of suction and thermal radiation. The governing partial differential equations (PDEs) have been subjected to a similarity transformation, resulting in a set of nonlinear ordinary differential equations (ODEs). These ODEs were solved numerically utilizing the code of bvp4c in the software of Matlab which offers high accuracy (4th order). The employed nanofluid model incorporates the effects of Brownian motion and thermophoresis. The present study illustrates the graphical depictions of the impacts of different governing parameters, namely Hartmann (M) number, curvature (γ) parameter, Brownian motion ($N_{b}$) parameter, mass suction (S) parameter, thermal radiation (Rd) parameter, and thermophoresis ($N_{t}$) parameter, on heat transfer, flow, and mass transfer characteristics. Comprehensive determination and visual presentation of the coefficient of skin friction, local Nusselt number, and local Sherwood number were conducted for a range of estimates of applied parameters. Based on our examination, it has been determined that dual similarity solutions are present within a specific range of mass suction parameters. The relationship between the Casson parameter and various fluid dynamic properties, such as skin friction coefficient, heat transfer rate, and mass transfer rates, has been found to exhibit a decreasing trend. Furthermore, the stability analysis discovered that the first solution exhibits linear stability, whereas the second solution displays linear instability. Additionally, the motivation behind this study is to enhance industrial performance through the optimization of thermal power generation systems, thereby increasing their overall efficiency.

## 1 Introduction

Nanofluids exhibit enhanced characteristics, such as increased temporal stability, reduced flow passage obstruction, and enhanced conductivity of thermal, in contrast to simple base fluids due to nanoparticle inclusion within the fluid medium. Hence, these fluids exhibit a wide range of practical applications in various fields, including pharmacological delivery systems, solar collectors, diabetic treatment through peristaltic pumps, electronic cooling, pharmacological administration mechanisms, and atomic applications. The introduction of the nanofluid concept was pioneered by Choi and Eastman [1], who proposed the blending of a convection fluid with solid nano-particles of extremely small size. Meanwhile, Khan and Pop [2] were the early adopters of a mathematical way to examine the impacts of Brownian motion and thermophoresis of nanofluid on a stretched sheet. Furthermore, the examination of nanofluids in the context of flow over stretching/shrinking cylinders offers a captivating opportunity to examine the augmentation of mass and heat transfer phenomena. Buongiorno model [3] effectively represents the complex interaction among different forces occurring at the nanoscale. Nanoparticles disperse across the fluid medium due to Brownian motion caused by heat variations. In addition, nanoparticles move in response to thermal differences, a process known as thermophoresis, which happens when temperatures change. This movement helps to enhance heat transfer. Thermal control in electronics, solar collectors, and medical therapies are just a few of the many applications that engineers and researchers have investigated using the Buongiorno nanofluid model. Nanofluids, when added to these systems, may improve heat transfer rates and maximize efficiency. Applying the Buongiorno model to the phenomenon of expanding and contracting cylinders has been the subject of a large amount of scholarly work. For various solutions, Rana et al. [4] used Buongiorno's model and Nielsen's effective viscosity model together. They found that the Nusselt number and skin friction both rise noticeably with increasing nanoparticle concentration. Shatnawi et al. [5] extended the above concept for the hybrid nanofluid for Casson model. Zaimi et al. [6] expanded upon Buongiorno's model by applying it to a shrinking cylinder. Roşca et al. [7] employed the identical Buongiorno model to investigate the flow of nanofluid over a shrinking/stretching cylinder. Shatnawi et al. [8] and Abbas et al. [9] used the model for non-Newtonian flow. Usman et al. [10] utilized the Buongiorno model on an inclined plane and discovered solution.

Fluids can be primarily categorized into two groups: Newtonian and Non-Newtonian. Examples of Newtonian liquids contain water and engine oil, which exhibit a linear stress-strain relationship. On the other hand, non-Newtonian fluids such as honey, paste, and paint demonstrate a nonlinear stress-strain relationship. We frequently encounter these types of fluids in our everyday experiences. Therefore, the examination of these fluids holds significant importance. The current research endeavors will center on the examination of non-Newtonian fluid. Several mathematical models have been put forth to describe the phenomena of flow, heat transfer, and mass transfer in non-Newtonian fluids. Several fluid models have been proposed in the literature, including the Prandtl fluid model [11,12], the Casson fluid model [13], the Carreau fluid model [14], and the Maxwell fluid model [15]. Furthermore, it is worth noting that the blood of humans is classified as a type of Casson fluid. The study conducted by Shah et al. [16] examined the flow behavior of Casson nano-fluid over the stretching sheet, taking into consideration the influence of activation energy and chemical reaction. The investigation conducted by Oyelakin et al. [17] examined the behavior of gyrotactic micro-organisms within the flow of Casson nanofluid. Authors Reza et al. [18] employed analysis of finite difference to investigate the unsteady MHD flow of a Casson fluid. The slip conditions of unsteady flow Casson nanofluid on a surface were examined in the studies undertaken by Ghadikolaei et al. [19] and Jamshed et al. [20]. Abbas et al. [21] investigated the Casson hybrid nanofluid model numerically where it has been observed that “the skin friction of Casson nanofluid increased and temperature gradient declined due to the increment of solid nanoparticle concentration”. Ali et al. [22] employed a non-Newtonian model of micropolar nanofluid and observed that an increase in material parameters leads to a decrease in the compactness, concentration, and temperature. In their research articles, Dero et al. [23,24] investigated non-Newtonian models, revealing the presence of dual solutions within specific ranges of the applied parameters. In their investigation of the Casson fluid flow model, Lund et al. [25] identified dual solutions.

In the year 1942, Haanas Alfren made a significant contribution to the field of MHD by introducing relevant terminology. Numerous scholars have conducted extensive research to comprehend the characteristics of MHD and examine its effects on various aspects of nanofluid. Presently, this phenomenon finds applications in diverse domains including astrophysics, medical science, geography, and numerous others. MHD is a field of study that explores the interplay between fluid dynamics and magnetic fields. Analogies of this nature are conducted for fluids that possess electrical conductivity and lack magnetic properties. These fluids include hot ionized gases, strong electrolytes, and liquid metals. The analysis of the flow of MHD has extensive utilizations in various fields including engineering, industry, and technology. These applications encompass generators of MHD, thin magnetic fluid film stabilization, electric motors, transformers, mass spectrometers for designing cooling systems, and more. The manipulation of the magnetic field allows for the modification of numerous physical properties exhibited by such materials. The study conducted by Hayat et al. [26] examines the influence of the activation energy of Arrhenius on a nonlinear stretched sheet in the existence of convective third-grade nanofluid in MHD flow. The treatment of nanomaterials, irrespective of the presence of MHD streamline, has been examined in the context of a melting sheet by Dinh et al. [27]. The examination accomplished by Rashid et al. [28] studied the flow of MHD nanofluid on the formation of porous with shrunk walls of entropy. The research carried out by Pavar et al. [29] inspected the MHD current of nanofluid flowing semi-infinitely through a vertical permeable plate. In their study, Turkyilmazoglu [30] investigated the MHD flow that arises from a rotating disc undergoing a shrinking process. The flow behavior of nanofluid in the existence of the field of magnetic was investigated by Nagaraju et al. [31] via the ramped surface model. Abbas and Shatanawi [32] investigated the effects of Brownian motion and thermophoresis on non-Newtonian fluids. The same Buongiorno model was extended for temperature-dependent non-Newtonian fluid flow over the Riga sheet [33]. In their study, Kumar et al. [34] investigated the influence of MHD effect on Casson nanofluid, incorporating considerations for thermal radiation and Hall current. In their study, Azmi et al. [35] expanded upon the MHD concept to encompass Casson fluid, investigating the implications of first-order slip conditions. In their study, Mahabaleshwar et al. [36] explored the axisymmetric flow of a non-Newtonian fluid, examining the influence of MHD on the process. Their findings revealed a positive impact of MHD on heat transfer within the system. In their study, Lund et al. [37] incorporated the MHD effect while investigating the behavior of Casson fluid over a vertical surface. The findings revealed that the Hartman number exhibited opposite directions in both solutions.

The latest and intriguing article provides a compilation of significant references about MHD flow on diverse flow geometries [[34], [35], [36], [37]].

The significance of convective heat transfer in various industrial and environmental applications, such as gas turbines, energy storage, photovoltaic panels, geothermal reservoirs, nuclear plants, and rocket propulsion, has generated considerable attention. The convective boundary condition (BC) has garnered attention in the literature, often being modeled using a Biot number within the thermal BC of the wall. Furthermore, a Biot number less than one signifies that the conduction resistance within the fluid is significantly smaller in comparison to the convection resistance at the surface. The Biot number is defined as unity, indicating that both conduction within the fluid and convection at the surface exhibit similar levels of significance. A Biot number greater than one directs that conduction resistance within an object is substantially higher than convection resistance at a sheet. In the latest examination undertaken by Shahzad et al. [38], authors examined the similarity solutions of the flow and heat transfer characteristics of power law fluid on the stretched cylinder. The analysis also incorporated the effect of a convective BC. In their study, Rashad et al. [39] communicated a similarity solution for convective surface BC of the Williamson hybrid nanofluid. Aziz [40] conducted a study on the characteristics of thermal and hydrodynamic slip flow boundary layers, employing a temperature BC. In their study, Gupta et al. [41] employed a variational finite element method to model the behavior of radiative mixed convective micropolar flow on shrunk surface. The simulation incorporated a convective BC. Analysis undertaken by Bég et al. [42] examined the influence of buoyancy and Biot number effects on MHD slip flows.

This research paper focuses on investigating the MHD flow of a Casson nanofluid around a permeable cylinder that undergoes stretching or shrinking. Enhanced thermal conductivity, reduced friction, and exceptional stability are just a few of the benefits of using Casson nanofluids for heat transfer and viscosity controlling. This research has real-world implications in many different areas, such as heat exchangers for industrial use, solar thermal systems, automobiles, and electronic cooling. Because these allow for controlled thermal treatments and medical imaging, these fluids are vital to biomedical devices, which are ideal for many uses for controlled and efficient heat transfer because of their impressionability in rheological characteristics. Current research mathematical model also depends totally on Buongiorno's [3] nanofluid. It is important to note that Hayat et al. [43], Bakar et al. [44], and Roşca et al. [45] have investigated the effect of radiation on nanofluid over the permeable cylinder during stretching and shrinking. It should be emphasized that, as far as we are aware, the investigation of stretching/shrinking cylinder in a magnetized Casson nanofluid for multiple solutions with stability analysis, utilizing the mathematical nanofluid model introduced by Buongiorno, was not previously explored. Hence, the prime aims of the current investigation endeavor are to examine the influence of MHD and Biot number on Casson nanofluid, anticipate the presence of multiple solutions, and conduct a stability analysis to ascertain the solution that exhibits stability.

## 2 Mathematical modeling

This study focuses on analyzing the characteristics of a specific fluid flow under following assumptions.

**Fig. 1:**
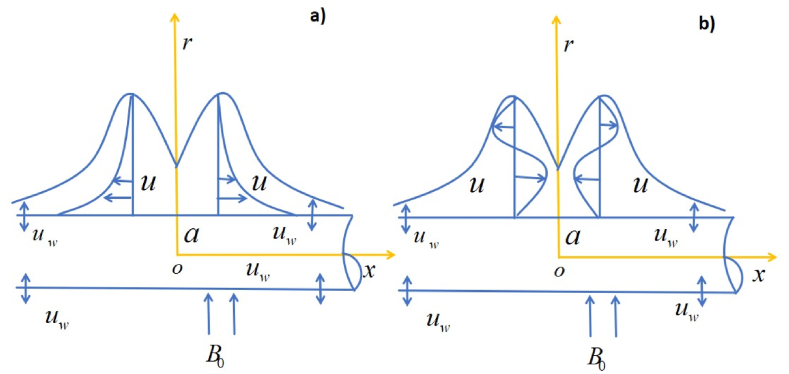
(a, b): Physical model consists of two cylinders: one that stretches (a) and one that shrinks (b).

1. Flow is the incompressible, steady flow of Casson nanofluid.
2. Surface is stretching/shrinking cylinder of radius R along with velocity $U_{w}=\frac{cx}{L}$, where c is continuous with c<0 corresponding to shrunk constant and c>0 corresponding to stretched constant, x is coordinate with cylinder, and L is the characteristic length.
3. Mass suction is assumed as $v_{w}=-\frac{a}{r}\sqrt{\frac{c\vartheta }{L}}S$, where S>0 denotes suction and S<0 denotes injection.
4. Temperature of surface is $T_{f}$ , and $T_{\infty }$ is ambient temperature, where $T_{f}>T_{\infty }$.
5. The magnetic field is oriented perpendicular to the direction of the fluid's movement (see Fig. 1 (a, b)).

The governing boundary layer equations can be expressed using a modified version of Buongiorno's equations [3] as follow [[43], [44], [45]].

(1) $$\frac{\partial \left(ru\right)}{\partial x}+\frac{\partial \left(rv\right)}{\partial r}=0$$

(2) $$u\frac{\partial u}{\partial x}-\left(\vartheta +\frac{\vartheta }{\beta }\right)\left\{\frac{\partial^{2}u}{\partial r^{2}}+\frac{1}{r}\frac{\partial u}{\partial r}\right\}+v\frac{\partial u}{\partial r}=-\;\frac{\sigma B_{0}^{2}u}{\rho }$$

(3) $$u\;\frac{\partial T}{\partial x}+v\;\frac{\partial T}{\partial r}=\alpha \left\{\frac{\partial^{2}T}{\partial r^{2}}+\frac{1}{r}\frac{\partial T}{\partial r}\right\}-\frac{1}{{\left(\rho c\right)}_{f}r}\frac{\partial \left(rq_{r}\right)}{\partial r}+\tau_{w}\;\left[D_{B}\;\frac{\partial C}{\partial r}\;\frac{\partial T}{\partial r}+\frac{D_{T}}{T_{\infty }}{\;\left(\frac{\partial T}{\partial r}\right)}^{2}\right]$$

(4) $$u\;\frac{\partial C}{\partial x}-D_{B}\left\{\frac{\partial^{2}C}{\partial r^{2}}+\frac{1}{r}\frac{\partial C}{\partial r}\right\}+v\;\frac{\partial C}{\partial r}=\frac{D_{T}}{T_{\infty }}\;\left[\frac{\partial^{2}T}{\partial r^{2}}+\frac{1}{r}\frac{\partial T}{\partial r}\right]$$

where u and v are the associated components of velocity, and r and x are the coordinates measured along the radial direction and cylinder's surface, respectively. Next, where denotes electrical conductivity, $B_{0}$ denotes the applied magnetic field, T denotes the boundary layer temperature, C denotes the concentration of nanoparticles therein, ϑ denotes kinematic viscosity, $D_{B}$ denotes Brownian diffusion, and $D_{T}$ denotes thermophoresis diffusion. In addition, $\alpha =\frac{k}{{\left(\rho c\right)}_{f}}$, where $\rho_{f}$ is the fluid density, $\rho_{p}$ is the particle density, and c is the coefficient of volumetric expansion. As for the BCs (refer to Hayat et al. [43] and Roşca et al. [45]),

(5) $$\left\{ \begin{array}{c} u=\lambda U_{w}\left(x\right),v=v_{w},k\left(\frac{\partial T}{\partial r}\right)=h_{f}\left(T_{f}-T\right),C=C_{w}\;at\;r=a\\ u\to 0,T\to T_{\infty },C\to C_{\infty }\;at\;r\to \infty \end{array}\right.$$

Under the stretching/shrinking cylinder, a flow with a coefficient of convective heat transfer $h_{f}$ and a temperature $T_{f}$ is present. By applying the similarity transformations, we search for solutions in similarity to Eqs. (1), (2), (3), (4) subject to BCs (5). It is important to acknowledge that within the field of mathematical modeling, transformations are employed to convert a system into an approximate system. Similarly, similarity transformations are utilized to decrease the number of independent and dependent variables in a given system of equations. In this context, we seek a suitable similarity transformation as proposed by Roşca et al. [45].

(6) $$\left\{ \begin{array}{c} v=-\frac{a}{r}\sqrt{\frac{c\vartheta }{L}}f\left(\eta \right),u=\frac{cx}{L}f^{\prime }\left(\eta \right)\\ \theta \left(\eta \right)=\frac{T-T_{\infty }}{T_{f}-T_{\infty }},\emptyset \left(\eta \right)=\frac{C-C_{\infty }}{C_{w}-C_{\infty }},\eta =\sqrt{\frac{c}{\vartheta L}}\frac{r^{2}-a^{2}}{2a}\; \end{array}\right.$$

where η is the similarity variable. The BCs at r=a are transformed into BCs at η=0, which simplifies the numerical calculations.

The following nonlinear ODEs are obtained by putting Eq. (6) into Eqs (2), (3), (4).

(7) $$\left(1+\frac{1}{\beta }\right)\left\{\left(1+2\gamma \eta \right)f^{\prime \prime \prime }+2\gamma f^{\prime \prime }\right\}+ff^{\prime \prime }-{f^{\prime }}^{2}-Mf^{\prime }=0$$

(8) $$\frac{1}{\mathrm{Pr}}\left(1+\frac{4}{3}Rd\right)\left\{\left(1+2\gamma \eta \right)\theta^{\prime \prime }+2\gamma \theta^{\prime }\right\}+f\theta^{\prime }+\left(1+2\gamma \eta \right)\left\{N_{b}\emptyset^{\prime }\theta^{\prime }+N_{t}{\left(\theta^{\prime }\right)}^{2}\right\}=0$$

(9) $$\left(1+2\gamma \eta \right)\emptyset^{\prime \prime }+2\gamma \emptyset^{\prime }+Scf\emptyset^{\prime }+\left(1+2\gamma \eta \right)\frac{N_{t}}{N_{b}}\theta^{\prime \prime }+2\gamma \frac{N_{t}}{N_{b}}\theta^{\prime }=0$$

put under the influence of (5) BCs,

(10) $$\left\{ \begin{array}{c} f\left(0\right)=S,f^{\prime }\left(0\right)=\lambda ,\theta^{\prime }\left(0\right)=Bi\left(\theta \left(0\right)-1\right),\emptyset \left(0\right)=1\\ f^{\prime }\left(\eta \right)\to 0,\theta \left(\eta \right)\to 0,\emptyset \left(\eta \right)\to 0\;\text{as}\;\eta \to \infty \end{array}\right.$$

For a given set of values of the other parameters (curvature parameter γ, Hartman number M, Casson parameter β, Prandtl number Pr, thermophoresis parameter $N_{t}$, Brownian motion parameter $N_{b}$, Schmidt number Sc, shrinking cylinder λ<0, stretching cylinder λ>0), this equation holds.

(11) $$\left\{ \begin{array}{c} \gamma =\sqrt{\frac{\vartheta L}{ca^{2}}}M=\frac{\sigma LB_{0}^{2}}{\rho c},Sc=\frac{\vartheta }{D_{B}},Bi=\frac{h_{f}r}{ka}{\left(\frac{\vartheta L}{c}\right)}^{0.5}\;\\ N_{t}=\frac{\tau_{w}D_{T}\left(T_{f}-T_{\infty }\right)}{\vartheta T_{\infty }},N_{b}=\frac{\tau_{w}D_{B}\left(C_{w}-C_{\infty }\right)}{\vartheta },\mathrm{Pr}=\frac{\vartheta }{\alpha }\; \end{array}\right.$$

The skin friction coefficient $C_{f}$, the local Nusselt number ${Nu}_{x}$, and the local Sherwood number ${Sh}_{x}$ are the physical quantities of interest, and they are specified as

(12) $$C_{f}=\frac{\tau_{1}}{\rho U_{\infty }^{2}},{Nu}_{x}=\frac{xq_{w}}{k\left(T_{f}-T_{\infty }\right)},{Sh}_{x}=\frac{xq_{m}}{D_{B}\left(C_{w}-C_{\infty }\right)}\text{,}$$

where the shear stress on the wall is denoted by $\tau_{1}$, the local heat flow is denoted by $q_{w}$, and the local mass flux is denoted by $q_{m}$.

(13) $$\tau_{1}={\mu \left(1+\frac{1}{\beta }\right)\left(\frac{\partial u}{\partial r}\right)}_{r=a},q_{w}={-k\left(\frac{\partial T}{\partial r}\right)}_{r=a}+{\left(q_{r}\right)}_{r=a},q_{m}={-D_{B}\left(\frac{\partial C}{\partial r}\right)}_{r=a}$$

Above quantities are calculated using the similarity variables (6).

(14) $${Re}_{x}^{1/2}C_{f}=\left(1+\frac{1}{\beta }\right)f^{\prime \prime }\left(0\right);\;{Re}_{x}^{-1/2}{Nu}_{x}=-\left(1+\frac{4}{3}Rd\right)\theta^{\prime }\left(0\right),{Re}_{x}^{-1/2}{Sh}_{x}=-\emptyset^{\prime }\left(0\right)$$

where ${Re}_{x}=\frac{U_{\infty }x}{\vartheta }$ is the local Reynolds number.

## 3 Stability analysis

In cases when there are more than two possible solutions, a stability analysis must be conducted. Dual solutions have been obtained in this research. In order to ensure the reliability of the solution, it is necessary to perform a stability analysis of Equations (7), (8), (9) with respect to the boundary conditions (10). We have modified Equations (2), (3), (4) regulating the unsteady flow by adding a time-dependent variable, denoted by $\tau =\frac{c}{L}.t$.

(15) $$\frac{\partial u}{\partial t}+u\frac{\partial u}{\partial x}-\left(\vartheta +\frac{\vartheta }{\beta }\right)\left\{\frac{\partial^{2}u}{\partial r^{2}}+\frac{1}{r}\frac{\partial u}{\partial r}\right\}+v\frac{\partial u}{\partial r}=-\;\frac{\sigma B_{0}^{2}u}{\rho }$$

(16) $$\frac{\partial T}{\partial x}+u\;\frac{\partial T}{\partial x}+v\;\frac{\partial T}{\partial r}=\alpha \left\{\frac{\partial^{2}T}{\partial r^{2}}+\frac{1}{r}\frac{\partial T}{\partial r}\right\}-\frac{1}{{\left(\rho c\right)}_{f}r}\frac{\partial \left(rq_{r}\right)}{\partial r}+\tau_{w}\;\left[D_{B}\;\frac{\partial C}{\partial r}\;\frac{\partial T}{\partial r}+\frac{D_{T}}{T_{\infty }}{\;\left(\frac{\partial T}{\partial r}\right)}^{2}\right]$$

(17) $$\frac{\partial C}{\partial t}+u\;\frac{\partial C}{\partial x}-D_{B}\left\{\frac{\partial^{2}C}{\partial r^{2}}+\frac{1}{r}\frac{\partial C}{\partial r}\right\}+v\;\frac{\partial C}{\partial r}=\frac{D_{T}}{T_{\infty }}\;\left[\frac{\partial^{2}T}{\partial r^{2}}+\frac{1}{r}\frac{\partial T}{\partial r}\right]$$

By introducing a new time-dependent variable into the transformation, we may rewrite Equation (6) as [44]:

(18) $$\left\{ \begin{array}{c} v=-\frac{a}{r}\sqrt{\frac{c\vartheta }{L}}f\left(\eta ,\tau \right),u=\frac{cx}{L}\frac{\partial f\left(\eta ,\tau \right)}{\partial \eta },\tau =\frac{c}{L}.t\\ \theta \left(\eta ,\tau \right)=\frac{T-T_{\infty }}{T_{f}-T_{\infty }},\emptyset \left(\eta ,\tau \right)=\frac{C-C_{\infty }}{C_{w}-C_{\infty }},\eta =\sqrt{\frac{c}{\vartheta L}}\frac{r^{2}-a^{2}}{2a}\; \end{array}\right.$$

when Equations (15), (16), (17) are modified by inserting (18), the resulting equations are as follows:

(19) $$\left(1+\frac{1}{\beta }\right)\left\{\left(1+2\gamma \eta \right)\frac{\partial^{3}f}{\partial \eta^{3}}+2\gamma \frac{\partial^{2}f}{\partial \eta^{2}}\right\}+f\frac{\partial^{2}f}{\partial \eta^{2}}-{\left(\frac{\partial f}{\partial \eta }\right)}^{2}-M\frac{\partial f}{\partial \eta }-\frac{\partial^{2}f}{\partial \tau \partial \eta }=0$$

(20) $$\frac{1}{\mathrm{Pr}}\left(1+\frac{4}{3}Rd\right)\left\{\left(1+2\gamma \eta \right)\frac{\partial^{2}\theta }{\partial \eta^{2}}+2\gamma \frac{\partial \theta }{\partial \eta }\right\}+f\frac{\partial \theta }{\partial \eta }+\left(1+2\gamma \eta \right)\left\{N_{b}\frac{\partial \emptyset }{\partial \eta }\frac{\partial \theta }{\partial \eta }+N_{t}{\left(\frac{\partial \theta }{\partial \eta }\right)}^{2}\right\}-\frac{\partial \theta }{\partial \tau }=0$$

(21) $$\left(1+2\gamma \eta \right)\frac{\partial^{2}\emptyset }{\partial \eta^{2}}+2\gamma \frac{\partial \emptyset }{\partial \eta }+Scf\frac{\partial \emptyset }{\partial \eta }+\left(1+2\gamma \eta \right)\frac{N_{t}}{N_{b}}\frac{\partial^{2}\theta }{\partial \eta^{2}}+2\gamma \frac{N_{t}}{N_{b}}\frac{\partial \theta }{\partial \eta }-\frac{\partial \emptyset }{\partial \tau }=0$$

with “BCs

(22) $$\left\{ \begin{array}{c} f\left(0,\tau \right)=S;\;\frac{\partial f\left(0,\tau \right)}{\partial \eta }=\lambda ;\;\theta \left(0,\tau \right)=1;\;\frac{\partial \theta }{\partial \eta }\left(0,\tau \right)=Bi\left(\theta \left(0,\tau \right)-1\right)\emptyset \left(0,\tau \right)=1\\ \frac{\partial f\left(\eta ,\tau \right)}{\partial \eta }\to 0;\;\theta \left(\eta ,\tau \right)\to 0;\emptyset \left(\eta ,\tau \right)\to 0\;as\;\eta \to \infty \end{array}\right.$$

With the following functions formulated (see Lund et al. [46]), we may test if the steady-state flow solutions $f\left(\eta \right)=f_{0}\left(\eta \right),\theta \left(\eta \right)=\theta_{0}\left(\eta \right)$ and $\emptyset \left(\eta \right)=\emptyset_{0}\left(\eta \right)$ of Equations (7), (8), (9) are stable.

(23) $$\left\{ \begin{array}{c} f\left(\eta ,\tau \right)=f_{0}\left(\eta \right)+e^{-\varepsilon \tau }\;F\left(\eta ,\tau \right)\\ \theta \left(\eta ,\tau \right)=\theta_{0}\left(\eta \right)+e^{-\varepsilon \tau }\;G\left(\eta ,\tau \right)\\ \emptyset \left(\eta ,\tau \right)=\emptyset_{0}\left(\eta \right)+e^{-\varepsilon \tau }\;H\left(\eta ,\tau \right) \end{array}\right.$$

As” compared to $f_{0}\left(\eta \right),\theta_{0}\left(\eta \right)$ and $\emptyset_{0}\left(\eta \right)$; F(η,τ), G(η,τ), and H(η,τ) are all quite little. The value of ε, the missing eigenvalue, has to be determined. Putting (23) into Eqs. (19), (20), (21) in place of τ=0 yields

(24) $$\left(1+\frac{1}{\beta }\right)\left\{\left(1+2\gamma \eta \right)F_{0}^{\prime \prime \prime }+2\gamma F_{0}^{\prime \prime }\right\}+f_{0}F_{0}^{\prime \prime }+F_{0}f_{0}^{\prime \prime }-2f_{0}^{\prime }F_{0}^{\prime }-MF_{0}^{\prime }+\varepsilon F_{0}^{\prime }=0$$

(25) $$\frac{1}{\mathrm{Pr}}\left(1+\frac{4}{3}Rd\right)\left\{\left(1+2\gamma \eta \right)G_{0}^{\prime \prime }+2\gamma G_{0}^{\prime }\right\}+f_{0}G_{0}^{\prime }+F_{0}\theta_{0}^{\prime }+\left(1+2\gamma \eta \right)N_{b}\left\{\emptyset_{0}^{\prime }G_{0}^{\prime }+H_{0}^{\prime }\theta_{0}^{\prime }\right\}+2\left(1+2\gamma \eta \right)N_{t}\theta_{0}^{\prime }G_{0}^{\prime }+\varepsilon G_{0}=0$$

(26) $$\left(1+2\gamma \eta \right)\left\{H_{0}^{\prime \prime }+\frac{N_{t}}{N_{b}}G_{0}^{\prime \prime }\right\}+2\gamma H_{0}^{\prime }+Sc\left\{f_{0}\emptyset_{0}^{\prime }+F_{0}H_{0}^{\prime }\right\}+2\gamma \frac{N_{t}}{N_{b}}G_{0}^{\prime }+Sc\varepsilon H_{0}=0$$

with the BCs

(27) $$\left\{ \begin{array}{c} F_{0}\left(0\right)=H_{0}\left(0\right)=F_{0}^{\prime }\left(0\right)=0,G_{0}^{\prime }\left(0\right)=BiG_{0}\left(0\right)\\ \;G_{0}\left(\eta \right)\;\to 0,F_{0}^{\prime }\left(\eta \right)\;\to 0,H_{0}\left(\eta \right)\to 0\;as\;\eta \to \infty \end{array}\right.$$

The stability of steady flow solution is determined by smallest eigenvalue $\varepsilon_{1}$, which depends on specific parameter values. This stability analysis is related to the steady solutions $f_{0}\left(\eta \right)$, $\theta_{0}\left(\eta \right)\text{,}$ and $\emptyset_{0}\left(\eta \right)$.

## 4 Numerical method

The system represented by equations (7), (8), (9) is solved through the utilization of the bvp4c procedure, employing the three-stage collocation formula. The collocation polynomial generated yields a consistently accurate fourth-order solution, maintaining $C^{1}$ continuity throughout the integration interval [44]. The collocation method employs a mesh to discretize the interval into smaller subintervals. The solver ensures the provision of a solution for the governing system. To evaluate the accuracy of the solution, a solver is applied to each subinterval to predict the error as explained in Ref. [45]. If the required tolerance is not met, the procedure is iterated with mesh adjustment. This method entails converting BVP into a corresponding IVP. As a result, we formed.

(28) $$f^{\prime }=F_{p},f^{\prime \prime }=F_{pp},\left(1+\frac{1}{\beta }\right)*\left\{\left(1+2*\gamma *\eta \right)*F_{pp}^{\prime }+2*\gamma *F_{pp}\right\}+F*F_{pp}-F_{p}*F_{p}-M*F_{p}=0$$

(29) $$\theta^{\prime }=\theta_{p},\emptyset^{\prime }=\emptyset_{p},\frac{1}{\mathrm{Pr}}*\left(1+\frac{4}{3}*Rd\right)*\left\{\left(1+2*\gamma *\eta \right)*\theta_{p}^{\prime }+2*\gamma *\theta_{p}\right\}+F*\theta_{p}+\left(1+2*\gamma *\eta \right)*\left\{N_{b}*\emptyset_{p}*\theta_{p}+N_{t}*\theta_{p}*\theta_{p}\right\}=0$$

(30) $$\emptyset^{\prime }=\emptyset_{p},\left(1+2*\gamma *\eta \right)*\emptyset_{p}^{\prime }+2*\gamma *\emptyset_{p}+Sc*F*\emptyset_{p}+\left(1+2*\gamma *\eta \right)*\frac{N_{t}}{N_{b}}*\theta_{p}^{\prime }+2*\gamma *\frac{N_{t}}{N_{b}}*\theta_{p}=0$$

along with BCs

(31) $$\left\{ \begin{array}{c} F\left(0\right)=S,F_{p}\left(0\right)=\lambda ,F_{pp}\left(0\right)=\alpha_{1}\;\\ \theta_{p}\left(0\right)=Bi*\left(\theta \left(0\right)-1\right),\theta_{p}\left(0\right)=\alpha_{2}\emptyset \left(0\right)=1,\emptyset_{p}\left(0\right)=\alpha_{3} \end{array}\right.$$

The system of first-order ODEs (28–31) is numerically solved by specifying initial points on a mesh and appropriately adjusting the initial approximations.

## 5 Findings and analysis

The governing ODEs (7–9) and corresponding BCs (10) were solved using the three-stage Lobatto III-A formula implemented in *bvp4c* code within Matlab software which offers high accuracy (4th order). Results obtained were subsequently compared with the findings reported in a previously published paper by Khashi'ie et al. [47] to assess the reliability of the methodology employed. The comparison revealed a significant similarity, as indicated in Table 1. This paper focuses on the characterization of velocity $f^{\prime }\left(\eta \right)$, temperature θ(η), and concentration ∅(η) profiles in the context of a shrinking cylinder, as it is important to note that multiple solutions can be observed in this scenario. In current consideration, we managed to obtain two different solutions within the given parameter ranges: $5\leq \beta \leq \infty ;S_{c}\leq S\leq 3;\;0\leq \gamma \leq 0.2;0.5\leq Bi\leq 2;\;0.1\leq N_{t}\leq 0.7;\;0.1\leq N_{b}\leq 0.7;\;0\leq Rd\leq 1\text{;}$ and 0.07≤M≤0.15. It should be noted that while the first solution is easily found by assigning actual boundary conditions, the trial-and-error basis technique was used to obtain the second solution in boundary conditions by assuming different guesses, subject to all boundary conditions being met.

**Table 1:** Contrast of $f^{\prime \prime }\left(0\right)$ for different magnitudes of S and γ when $Bi=Rd=0.5,\lambda =-1,M=0.1,\mathrm{Pr}=2,Sc=2,N_{b}=0.5=N_{t}\text{,}$ and β=∞.

γ		Khashi'ie et al. [47]		Current Results	
	S	$f^{\prime \prime }\left(0\right)$		$-\theta^{\prime }\left(0\right)$	$-\emptyset^{\prime }\left(0\right)$
0	2	1.31623 [0.68377]	1.316228 [0.68377]	0.36042 [0.35041]	3.13045 [2.93855]
	2.1	–	1.50000 [0.59989]	0.36827 [0.35684]	3.37572 [3.14108]
	2.2	–	1.65678 [0.54297]	0.37482 [0.36352]	3.60782 [3.35929]
0.1	2.1	–	1.41361 [0.88764]	0.36626 [0.35811]	3.33014 [3.16603]
	2.2	–	1.60079 [0.83744]	0.37357 [0.36385]	3.57648 [3.36822]
	2.3	–	1.75833 [0.82487]	0.37965 [0.37003]	3.80868 [3.58953]

[] second solution.

**Fig. 2:**
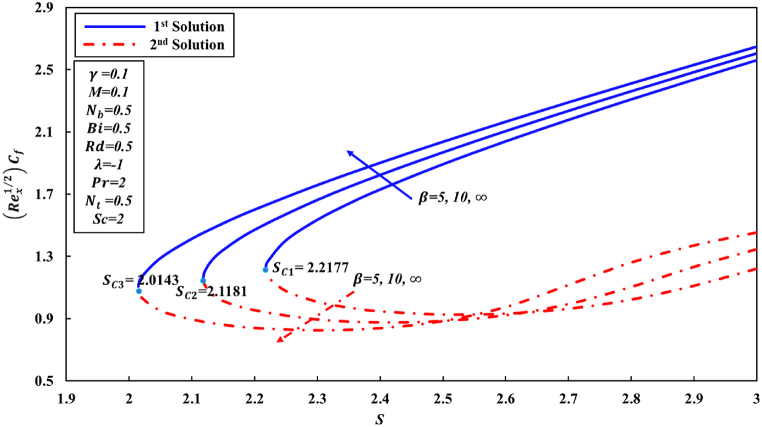
Changes to ${Re}_{x}^{1/2}C_{f}$ for various S and β values.

**Fig. 3:**
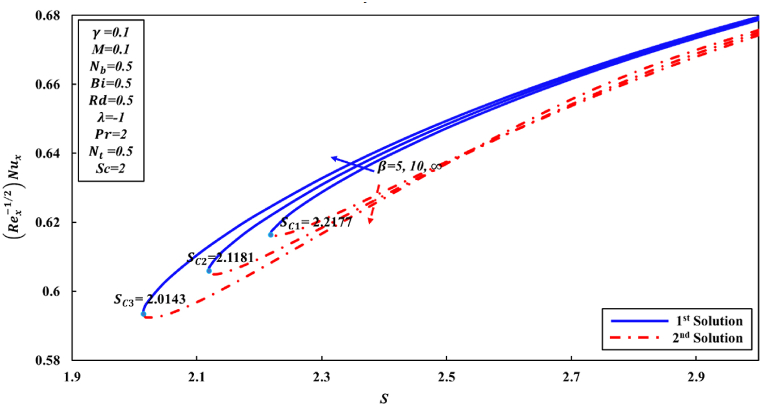
Changes to ${Re}_{x}^{-1/2}{Nu}_{x}$ for various Sandβ values.

**Fig. 4:**
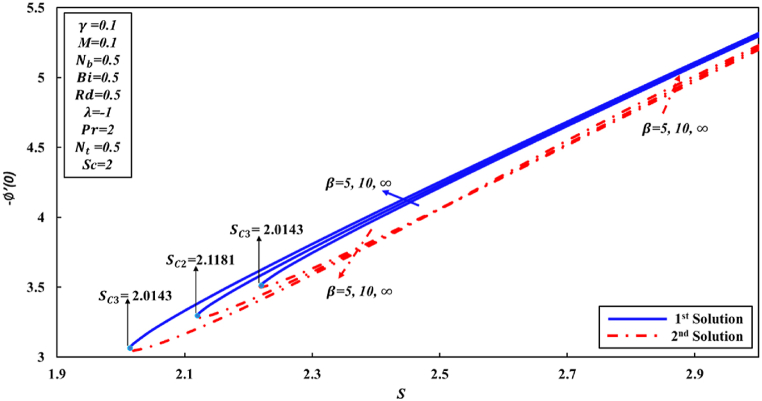
Changes to $-\emptyset^{\prime }\left(0\right)$ for various λandβ values.

According to Wang [48] and other researchers, it has been suggested in their analyses that the attainment of a similarity solution for problems of fluid flow on the shrunk sheet is feasible through the application of adequate wall mass suction. The behavior of Newtonian fluid is distinct from that of non-Newtonian fluid. In the case of Casson nanofluid, it has been observed that a significant mass suction is necessary to achieve a solution when the value of the Casson parameter β decreases. This study has revealed the presence of two distinct regions for similarity solutions, specifically dual solutions and no solution, which are contingent upon the mass suction parameter. For the values of β equal to 5, 10, and ∞ (representing the Newtonian fluid), a set of dual solutions is observed when S is greater than or equal to $S_{c1}$, $S_{c2}$, and $S_{c3}$, respectively. Conversely, a single similarity solution is present when S is less than $S_{c1}$, $S_{c2}$, and $S_{c3}$, as depicted in Fig. 2, Fig. 3, Fig. 4. When the level of suction is increased, skin friction coefficient $\left({Re}_{x}^{1/2}C_{f}\right)$ exhibits an increase in the first solution, while in the second solution, it initially experiences a decrease followed by an increase. Fig. 3, Fig. 4 were generated in order to investigate the impact of suction S and Casson parameter β on Nusselt number $\left({Re}_{x}^{-1/2}{Nu}_{x}\right)$ and Sherwood number $\left({Re}_{x}^{-1/2}{Sh}_{x}\right)$, correspondingly. In all graphical representations, the augmentation of suction results in an enhancement of both the Nusselt number and Sherwood number across all solutions.

**Fig. 5:**
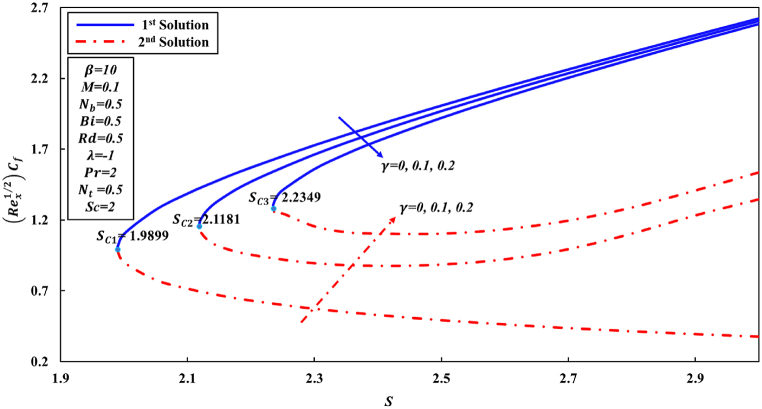
Changes to ${Re}_{x}^{1/2}C_{f}$ for various S and γ values.

**Fig. 6:**
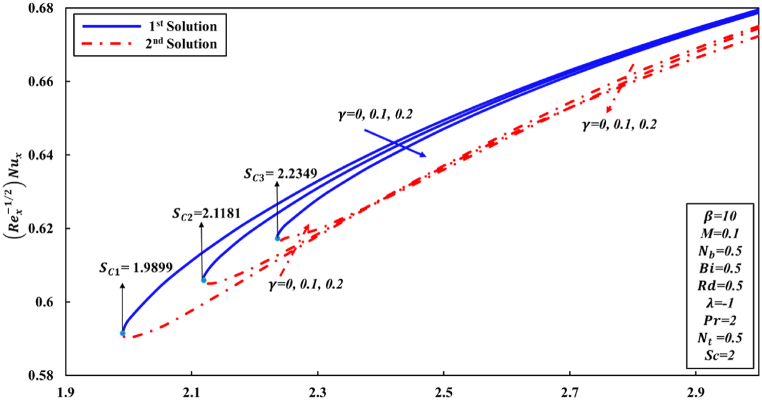
Changes to ${Re}_{x}^{-1/2}{Nu}_{x}$ for numerous S and γ values.

**Fig. 7:**
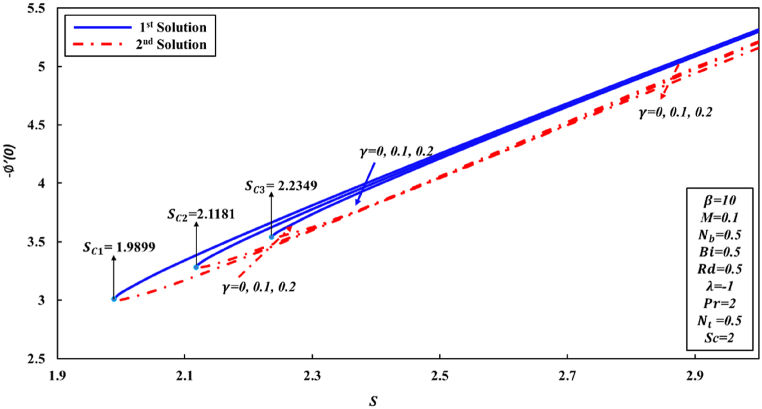
Changes to $-\emptyset^{\prime }\left(0\right)$ for different values of S and γ.

As, is already stated the skin friction coefficient (${Re}_{x}^{1/2}C_{f}$), Nusselt number $\left({Re}_{x}^{-1/2}{Nu}_{x}\right)$, and Sherwood number $\left({Re}_{x}^{-1/2}{Sh}_{x}\right)$ are physical quantities that are of great interest due to their significant engineering applications. According to the observations made in Fig. 5, it is followed that coefficient of skin friction tends to surge as the parameter S increases. Conversely, the value of ${Re}_{x}^{1/2}C_{f}$ reduces as curvature γ parameter increases in the first solution. This can be attributed to the fact that greater values of curvature γ parameter negatively impact the shear stress. The physical phenomenon occurs as a result of the decrease in parameter S, indicating to a decline in fluid velocity and a corresponding increase in friction force at the wall. Conversely, an inverse pattern is observed initially for the second solution. Conversely, it is observed that the ranges of dual solutions and the no solution can also be identified. Based on the analysis of Fig. 6, Fig. 7, it can be inferred that for γ values of 0, 0.1, and 0.2, there exists a range of dual solutions when the value of S is greater than or equal to $S_{c1}$, $S_{c2}$, and $S_{c3}$, respectively. Conversely, no similarity solution is observed when S is less than $S_{c1}$, $S_{c2}$, and $S_{c3}$, respectively. Fig. 7 illustrates the fluctuations in the gradient of concentration ∅(η) profile across several values of S and γ. It is observed that rate of concentration transfer for both solutions exhibits an increase as the value of S is incremented.

**Fig. 8:**
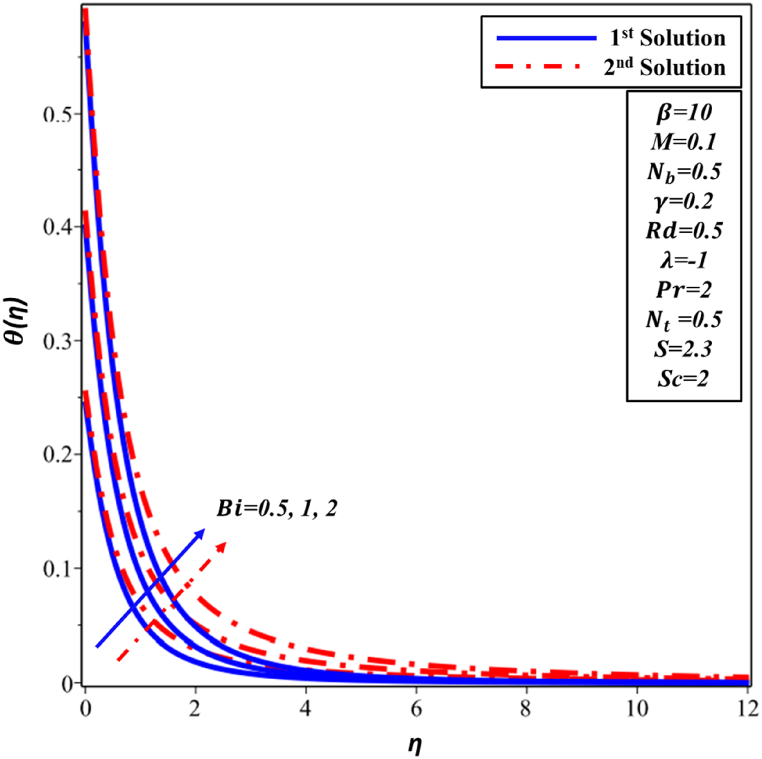
Changes in θ(η) against η for different values of Bi.

**Fig. 9:**
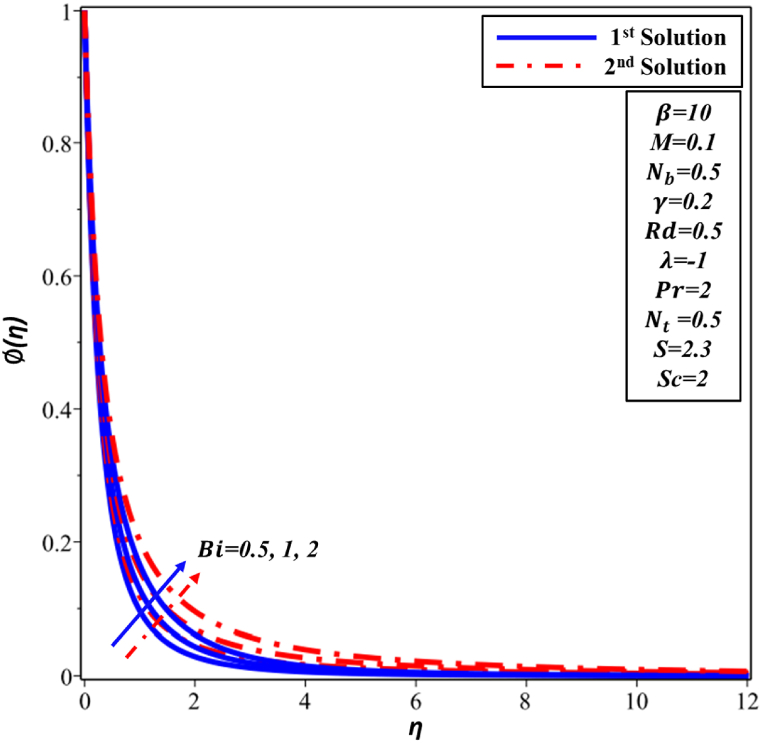
Changes in ∅(η) against η for different values of Bi.

Fig. 8 illustrates the fluctuations observed in the Biot number (Bi) across the temperature profile θ(η). The results clearly demonstrate that a increase in Biot number outcomes in an augmentation of both θ(η) distribution and thermal boundary layer (TBL) for both solutions. In practical terms, the feasibility arises from the relationship involving the Biot number, which quantifies the relationship between internal and external thermal resistances. As Biot number improves, the external thermal resistance decreases, leading to a thicker TBL. Fig. 9 depicts the concentration profile ∅(η) corresponding to varying Biot numbers. It has been observed that there is an increase in concentration in both the first and second solutions. From a physical standpoint, it can be argued that an elevated Biot number has a positive impact on convective heat transfer, facilitating improved dispersion and mixing of nanoparticles in the nanofluid. Consequently, this results in the increased ∅(η) profile.

**Fig. 10:**
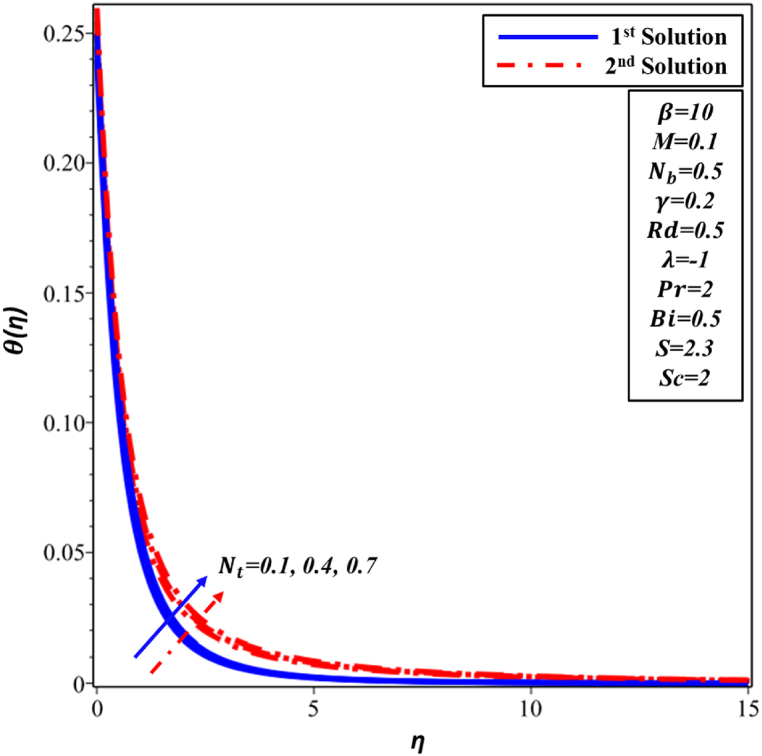
Changes in θ(η) against η for different values of $N_{t}$.

**Fig. 11:**
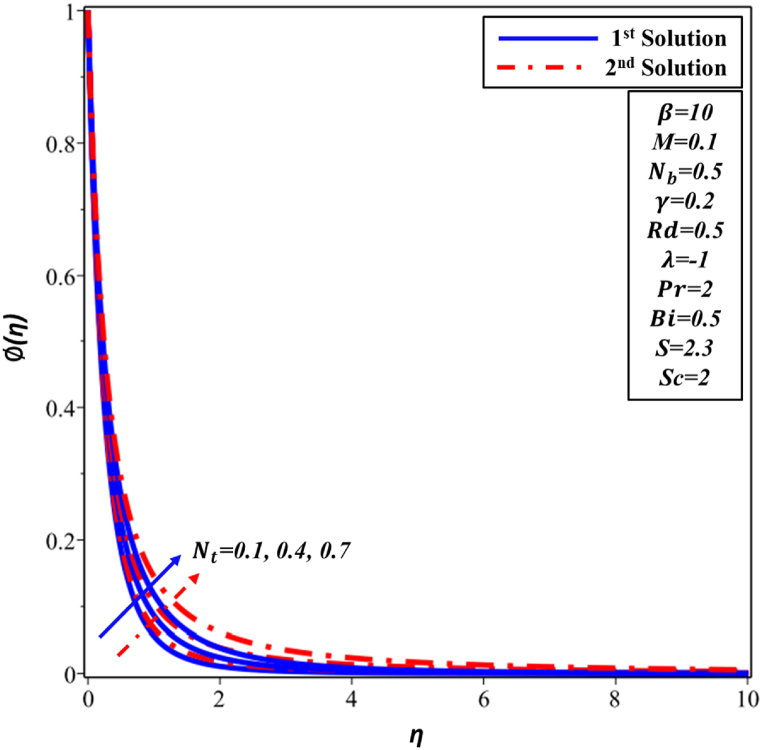
Changes in ∅(η) against η for different values of $N_{t}$.

Fig. 10 illustrates a positive correlation between θ(η) profile and thermophoresis parameter $N_{t}$ in both first and second solutions. The phenomenon that has been noticed can be explained by to $N_{t}$, and this is what propels the nanoparticles away from the surface and into the surrounding flow. Consequently, this leads to an increase in TBL. Fig. 11 illustrates the ∅(η) profile as $N_{t}$ increases. The concentration of the first and second solutions is observed to increase as the value of $N_{t}$ increases. The physical explanation for this phenomenon is that a large number of nanoparticles are moving toward the surrounding flow, which raises the concentration of these particles and causes the concentration boundary layer to grow.

**Fig. 12:**
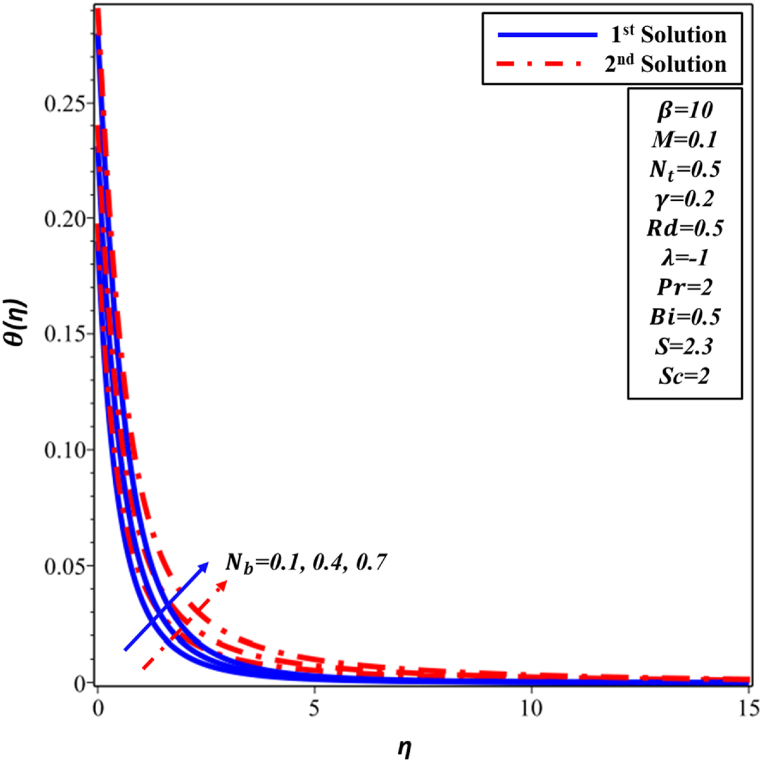
Changes in θ(η) against η for different values of $N_{b}$.

**Fig. 13:**
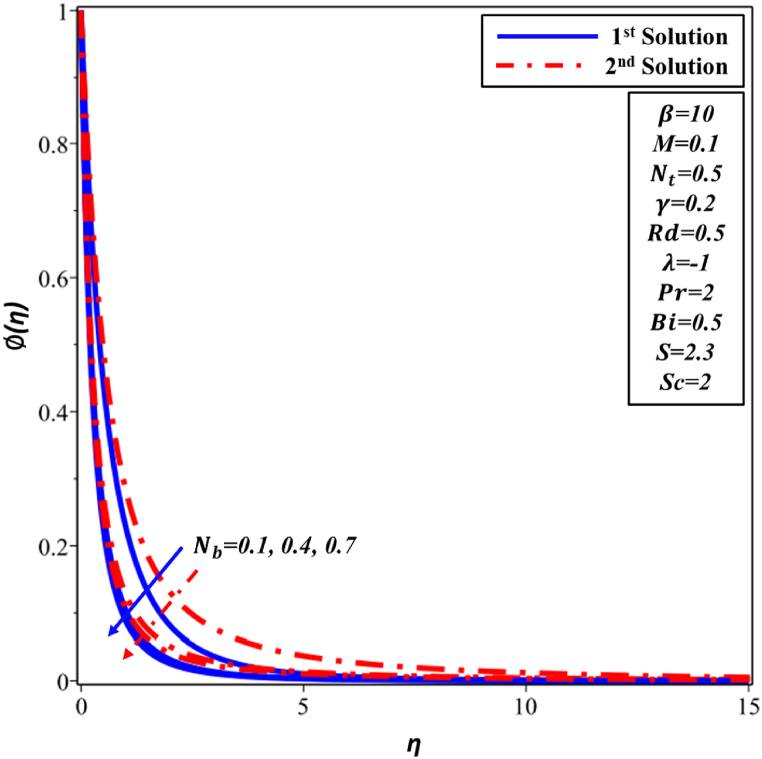
Changes in ∅(η) against η for different values of $N_{b}$.

Fig. 12 illustrates the variation of the Brownian motion $N_{b}$ with respect to the temperature profile θ(η). The relationship between the values of $N_{b}$ and fluid temperature in the first and second solutions exhibits a clear positive correlation. Nevertheless, the heightened activity of nanoparticles results in a rise in thickness of TBL. Consequently, these nanoparticles have a substantial effect on behavior of the boundary layer and induce alterations in the θ(η) distribution in far proximity to the surface. Fig. 13 illustrates the impact of $N_{b}$ on the concentration profile ∅(η). A decline in behavior has been noted in the initial and subsequent solutions as the values of $N_{b}$ increase. This phenomenon can be attributed to the correlation between the increased value of $N_{b}$ and the heightened random motion occurring within the boundary layer. Consequently, this augmented motion does not result in a proportional increase in resistance, thereby leading to a reduction in the concentration of nanoparticles.

**Fig. 14:**
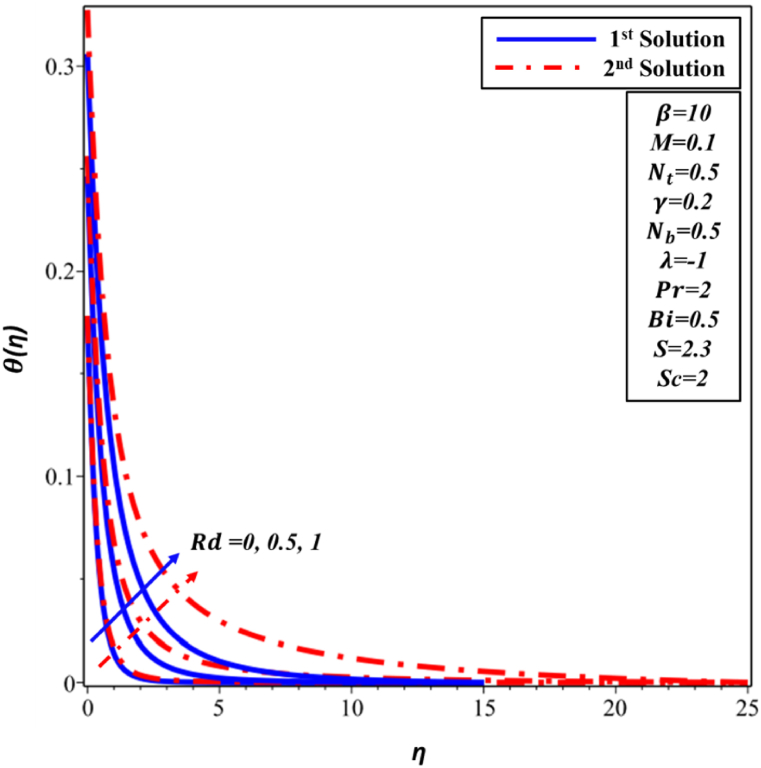
Changes in θ(η) against η for different values of Rd.

**Fig. 15:**
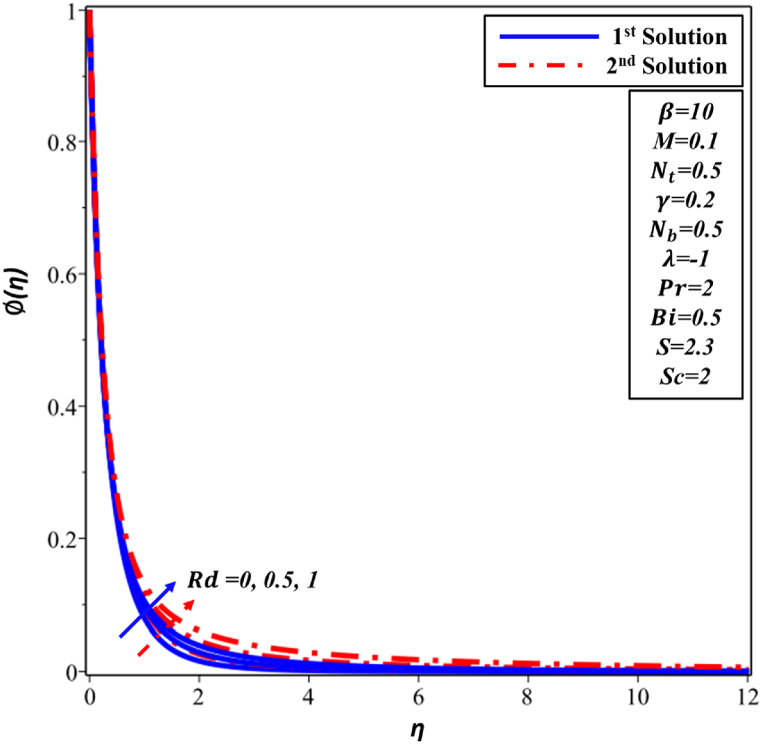
Changes in ∅(η) against η for different values of Rd.

The impact of thermal radiation (Rd) on the dimensionless temperature profile θ(η) is demonstrated in Fig. 14. θ(η) profile exhibits an increase as the enhancement of Rd occurs in both solutions. Nevertheless, as Rd raises, one sees a matching rise in the thickness of TBL in both solutions. The observed variations in the θ(η) distribution can be attributed to the influence of radiative heat transfer, specifically the net energy flux resulting from thermal radiation. According to Fig. 15, it can be observed that there is an increase in ∅(η) for solutions with larger values of Rd parameter in both the first and second solutions cases.

**Fig. 16:**
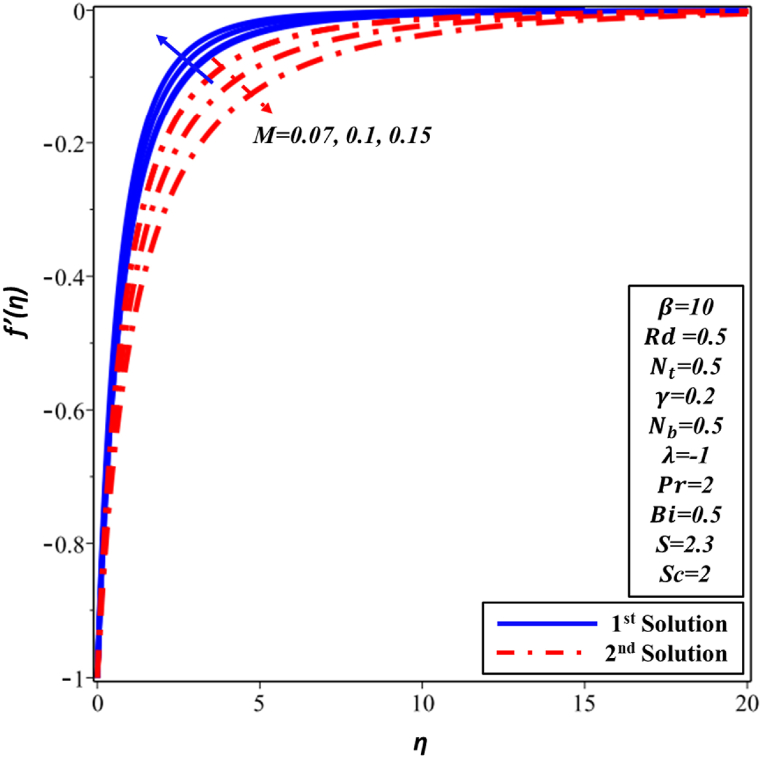
Changes in $f^{\prime }\left(\eta \right)$ against η for different values of M.

**Fig. 17:**
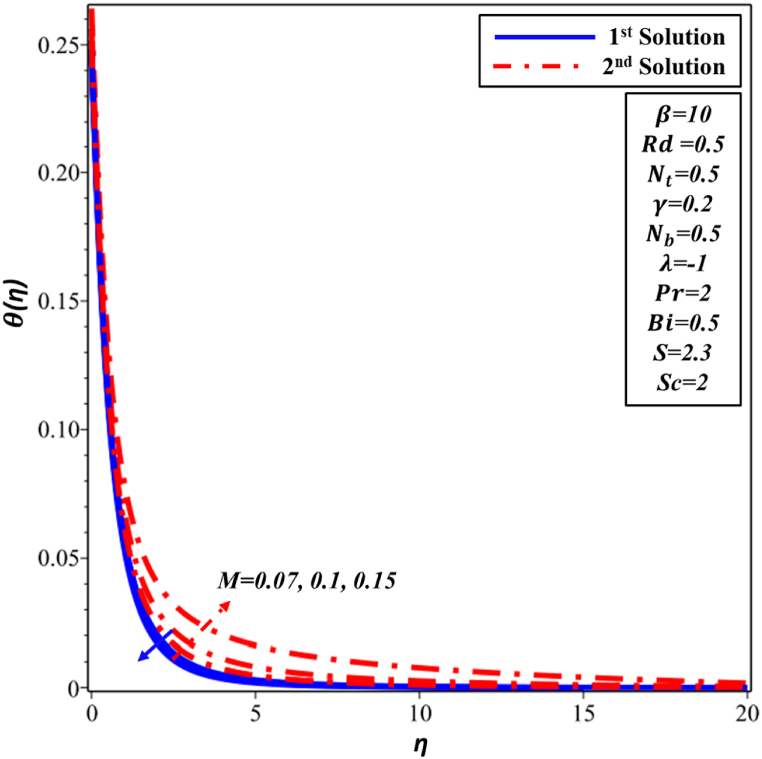
Changes in θ(η) against η for different values of M.

**Fig. 18:**
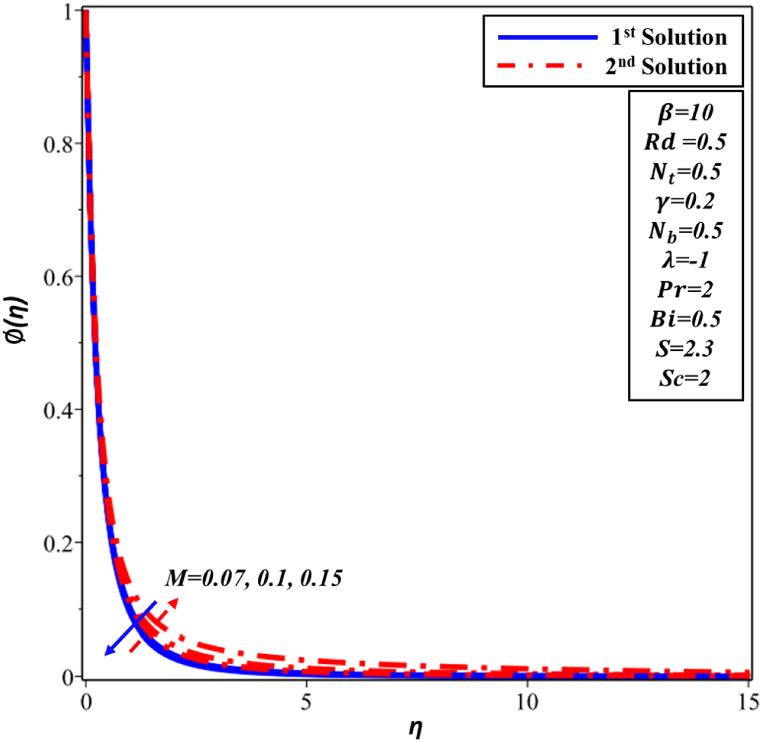
Changes in ∅(η) against η for different values of M.

The velocity profile $f^{\prime }\left(\eta \right)$ for various Hartman number M is depicted in Fig. 16. $f^{\prime }\left(\eta \right)$ is noticed to reduce as M magnitude rises in the first solution. The improvement in the intensity of the magnetic field results in the generation of a resistant force recognized as the Lorentz force, which signifies the deceleration of $f^{\prime }\left(\eta \right)$. Velocity and momentum boundary layer's thickness both exhibit an increase as the Hartmann number (M) is augmented in the second solution. The temperature profile θ(η), as depicted in Fig. 17, is presented in relation to the Hartmann number M. Second solution exhibits a direct correlation between the Hartmann number and both TBL thickness and the temperature. The increase in Hartmann number leads to heightened resistance within the boundary layer, resulting in an elevation of the nanofluid's temperature. The observed phenomenon in the first solution is the declining trend exhibited by θ(η) profile and TBL. The analysis of Fig. 18 reveals that the thickness of concentration decreases in the first solution as the Hartmann number M is increased. There is a lack of significant observable alteration. In contrast, the ∅(η) profile exhibits an increase as the Hartmann number M is enhanced in the second solution. This behavior can be attributed to the higher Hartmann numbers inducing more pronounced magnetic effects, which in turn alter the movement of nanoparticles and fluid flow, consequently modifying the ∅(η) profile of the nanofluid.

Table 2 presents the smallest eigenvalues ($\varepsilon_{1}$) for both solutions, considering various values of S and curvature parameter (γ). The elementary objective of our study is to assess the stability of the solutions in order to determine the stable solution among the two solutions. It is imperative to acknowledge that the physical interpretation of a solution is contingent upon its stability. From a mathematical perspective, it is essential to consider all potential multiple solutions, if they exist, as they are integral to the overall fluid flow problem and satisfy all necessary solution criteria. It is important to acknowledge that the stability of solution is contingent on the smallest eigenvalue sign. Findings presented in Table 2 demonstrate that $\varepsilon_{1}$ exhibits negative (positive) values. These values signify an initial growth (decay) of the disturbance, which in turn disrupts (resumes) the separation of the boundary layer and influences the stability (instability) of the flow. It is noteworthy to mention that the first solution, known as the stable solution, consistently yields a meaningful physical interpretation that can be observed.

**Table 2:** Minimum eigenvalue $\varepsilon_{1}$ for different combinations of S and γ, with $Bi=Rd=0.5,\lambda =-1,M=0.1,\mathrm{Pr}=2,Sc=2,N_{b}=0.5=N_{t}$ and β=10.

γ	S	$\varepsilon_{1}$	
		1st Solution	2nd Solution
0	1.99	0.0004	−0.0009
	2	0.0051	−0.0683
	2.2	0.3086	−0.3418
0.1	2.119	0.0009	−0.0006
	2.2	0.0926	−0.0987
	2.4	0.2767	−0.6576

## 6 Conclusion

This article exhibits an examination of the steady flow, heat transfer, and mass transfer characteristics of a magnetized Casson nanofluid over the stretched/Shrunk cylinder. The Buongiorno model has been modified to examine the influence of Rd, $N_{t}$ and $N_{b}$ effects. Viable transformations are employed in order to derive a set of simplified ODEs. The *bvp4c* solver function in Matlab software is employed to investigate the solutions of these ODEs. The following key findings are enumerated:

1. Dual solutions are observed exclusively on a shrinking cylinder.
2. There exists a range of dual solutions when the value of S is greater than or equal to $S_{c1}$, $S_{c2}$, and $S_{c3}$.
3. The inclusion of γ has a significant effect on profiles of $f^{\prime }\left(\eta \right)$, θ(η), and ∅(η).
4. Local Nusselt number exhibits an expanding trend with an upsurge in S, while it demonstrates a decreasing trend with an increase in γ.
5. First solution demonstrates an inverse correlation between the $f^{\prime }\left(\eta \right)$ profile and magnetic M parameter.
6. Profiles of temperature and concentration increase in both solutions for the rising effects of biot number.
7. The temperature and concentration profiles of both solutions exhibit an increase due to the escalating influence of thermal radiation.
8. Sherwood number exhibits a rising trend as the suction parameter is expanded in both the second and first solutions.
9. Stable solution refers to the first solution that satisfies the physical constraints.

The findings of current paper hold significant associations in the domain of heat and mass transfer, specifically in relation to the utilization of non-Newtonian Casson nanofluids. These results also present several promising avenues for further research. Casson nanofluids exhibit distinct characteristics that render them suitable for a wide range of applications, such as medical utilization in the context of blood flow, enhancement of thermal energy technologies within the energy sector, and utilization in solar energy systems to power aircraft and ships. Potential areas of investigation for future studies may encompass the examination of entropy generation, multi-scale analysis, experimental validation, micro-morphic constitutive, and hybrid nanofluid model.

## Data availability statement

The data that support the study's findings are available upon reasonable request from the corresponding author.

## Funding

“Project financed by 10.13039/501100015999Lucian Blaga University of Sibiu through research grant 10.13039/501100015999LBUS - IRG - 2023 - 09”.

## CRediT authorship contribution statement

**Azhar Mustafa Soomro:** Writing – original draft, Investigation, Formal analysis, Data curation, Conceptualization. **Mustafa Abbas Fadhel:** Writing – original draft, Software, Methodology, Investigation, Data curation. **Liaquat Ali Lund:** Visualization, Validation, Software, Methodology, Investigation. **Zahir Shah:** Writing – review & editing, Validation, Supervision, Methodology, Investigation, Conceptualization. **Mansoor H. Alshehri:** Writing – review & editing, Visualization, Project administration, Investigation, Formal analysis. **Narcisa Vrinceanu:** Writing – review & editing, Resources, Project administration, Investigation, Funding acquisition.

## Declaration of competing interest

The authors declare that they have no known competing financial interests or personal relationships that could have appeared to influence the work reported in this paper.
